# Tin dioxide nanomaterial-based photocatalysts for nitrogen oxide oxidation: a review

**DOI:** 10.3762/bjnano.13.7

**Published:** 2022-01-21

**Authors:** Viet Van Pham, Hong-Huy Tran, Thao Kim Truong, Thi Minh Cao

**Affiliations:** 1 Photocatalysis Research Group (PRG), Faculty of Materials Science and Technology, University of Science, VNU–HCM, 227 Nguyen Van Cu Street, District 5, Ho Chi Minh City, 700000, Viet Nam; 2 HUTECH University, 475A Dien Bien Phu Street, Binh Thanh District, Ho Chi Minh City, 700000, Viet Nam

**Keywords:** green products, nanomaterials, NO oxidation, photocatalysis, SnO_2_

## Abstract

Semiconducting SnO_2_ photocatalyst nanomaterials are extensively used in energy and environmental research because of their outstanding physical and chemical properties. In recent years, nitrogen oxide (NO*_x_*) pollutants have received particular attention from the scientific community. The photocatalytic NO*_x_* oxidation will be an important contribution to mitigate climate change in the future. Existing review papers mainly focus on applying SnO_2_ materials for photocatalytic oxidation of pollutants in the water, while studies on the decomposition of gas pollutants are still being developed. In addition, previous studies have shown that the photocatalytic activity regarding NO*_x_* decomposition of SnO_2_ and other materials depends on many factors, such as physical structure and band energies, surface and defect states, and morphology. Recent studies have been focused on the modification of properties of SnO_2_ to increase the photocatalytic efficiency of SnO_2_, including bandgap engineering, defect regulation, surface engineering, heterojunction construction, and using co-catalysts, which will be thoroughly highlighted in this review.

## Review

### Introduction

**Figure 1:**
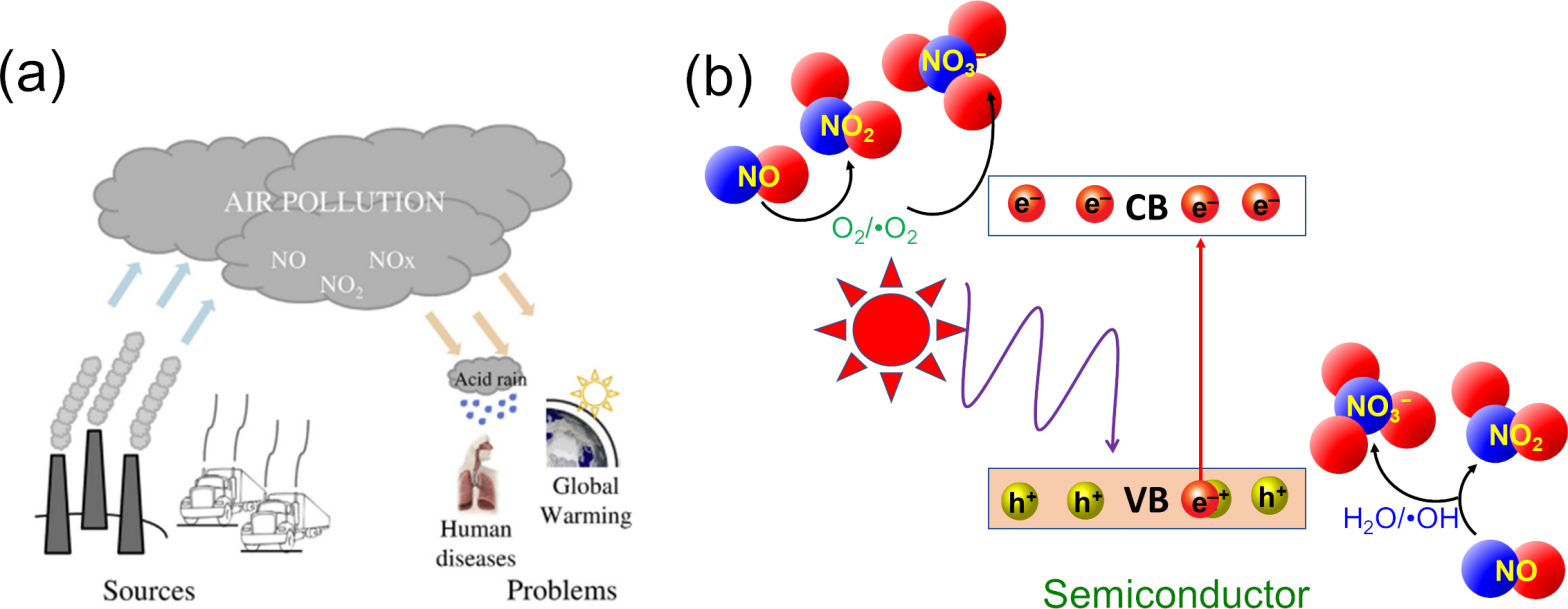
Problems and sources associated with NO*_x_* air pollution (a) and NO photocatalysis over a semiconductor (b). Figure 1a was reprinted from [12], Journal of Environmental Management, vol. 129, by J. Ângelo; L. Andrade; L. M. Madeira; A. Mendes “An overview of photocatalysis phenomena applied to NO_x_ abatement”, pages 522–539, Copyright (2013), with permission from Elsevier. This content is not subject to CC BY 4.0.

A World Health Organization (WHO) report indicated that 4.2 million deaths every year occur due to exposure to ambient (outdoor) air pollution [1]. This number is much higher than the deaths from the COVID-19 pandemic in the past year. WHO also reported that the emissions of nitrogen oxides in the early 1980s over the world were estimated at approximately 150 × 10^12^ g/year while the concentration of nitrogen dioxide outdoor can achieve up to 940 µg/m^3^ (0.5 ppm) for 30 min and 400 µg/m^3^ (0.21 ppm) for 60 min [2]. Nitrogen oxides (NO*_x_*, including NO and NO_2_) are poisonous and highly reactive gases. Nitrogen dioxide (NO_2_) is associated with respiratory diseases and mortality. NO*_x_* is formed when fuel is burnt at high temperatures and emitted by automobiles, trucks, and various non-road vehicles (e.g., construction equipment, boats) and industrial sources such as power plants, industrial boilers, cement kilns, and turbines [3]. In addition, diesel vehicles are considered a primary NO*_x_* emission source causing adversely impacts on environment and human health, such as acid rain, global warming, and respiratory diseases in humans (Figure 1a). NO*_x_* pollution damages lung cells and reacts with molecules in the air when released into the ozone layer. NO*_x_* can aggravate respiratory diseases such as asthma, bronchitis, and cardiovascular diseases. When humans are exposed to NO_2_ at concentrations of over 200 µg/m^3^, even for periods of time, this will cause adverse effects on the respiratory system. Some studies have shown that NO_2_ concentrations over 500 µg/m^3^ can cause acute health effects. Although the lowest threshold for NO_2_ exposure with a direct effect on lung function in asthmatic subjects was 560 µg/m^3^, NO_2_ exposure to concentrations over 200 µg/m^3^ caused pulmonary responses in asthmatic people [4–5]. Guillaume P. Chossière et al. indicated that reducing NO*_x_* in the air will significantly reduce the risk of death in humans demonstrated through a study on lockdowns during the COVID-19 pandemic in China that led to a reduction of NO_2_, O_3_, and PM_2.5_ concentrations globally, resulting in ca. 32,000 avoided premature mortalities, including ca. 21,000 in China [6]. Therefore, the control, treatment, and conversion of NO*_x_* to green products greatly interested the scientific community in recent years.

There are many methods for controlling and removing NO*_x_*, such as reducing the burning temperature, reducing the residence time at peak temperature, chemical reduction or oxidation of NO*_x_*, removal of nitrogen from combustion fuels, and sorption, both adsorption and absorption [7–8]. Among them, photocatalytic oxidation is an efficient method of converting NO*_x_* into nitrate (NO_3_^−^) ions. The removal of NO_3_^−^ ions is easy, efficient, and economic through chemical or biological methods such as the conversion of NO_3_^−^ to N_2_ by aerobic microorganisms [9–10]. Figure 1b illustrates the working scheme of semiconductor photocatalysts for NO oxidation. Light generates holes (h^+^) in the valence band (VB) and electrons (e^–^) in the conduction band (CB) of the photocatalytic material. Electrons at the material surface will react with oxygen molecules to form superoxide radicals (^•^O_2_^−^, similarly holes react with water to form hydroxyl radicals). Free radicals and strong oxidizing agents react with NO*_x_* to produce NO_3_^−^, deposited on the photocatalyst surface. The NO_3_^−^ product formed on the surface of the catalyst can be easily separated for further treatment by washing with water due [11] (see Equations 1–10).


[1] $$\text{Semiconductor}+h\nu \to \text{Semiconductor}\left({\text{h}}_{\text{VB}}^{+}+{\text{e}}_{\text{CB}}^{-}\right)$$

[2] $${\text{O}}_{2}+{\text{e}}_{\text{CB}}^{-}\to {\;}^{•}{\text{O}}_{2}^{-}$$

[3] $${\text{H}}_{2}\text{O}+{\text{h}}_{\text{VB}}^{+}\to {\text{HO}}^{•}+{\text{H}}^{+}$$

[4] $${}^{•}{\text{O}}_{2}^{-}+{\text{H}}_{2}\text{O}\to {\text{HO}}_{2}^{•}+{\text{OH}}^{-}$$

[5] $$2{\;}^{•}{\text{HO}}_{2}\to {\text{O}}_{2}+{\text{H}}_{\text{2}}{\text{O}}_{2}$$

[6] $${\text{HO}}_{2}^{•}+{\text{H}}_{2}\text{O}+{\text{e}}_{\text{CB}}^{-}\to {\text{H}}_{2}{\text{O}}_{2}+{\text{OH}}^{-}$$

[7] $${\text{2}}^{•}{\text{O}}_{2}^{-}+2{\text{H}}_{2}\text{O}\to 2{\text{H}}_{2}{\text{O}}_{2}+{\text{O}}_{2}$$

[8] $${\text{H}}_{2}{\text{O}}_{2}+{\text{e}}_{\text{CB}}^{-}\to {\;}^{•}\text{OH}+{\text{OH}}^{-}$$

[9] $$\text{NO}+{\;}^{•}{\text{O}}_{2}^{-}\to {\text{NO}}_{3}^{-}$$

[10] $$\text{NO}+2{\text{HO}}^{•}\to {\text{NO}}_{2}+{\text{H}}_{2}\text{O}$$

**Figure 2:**
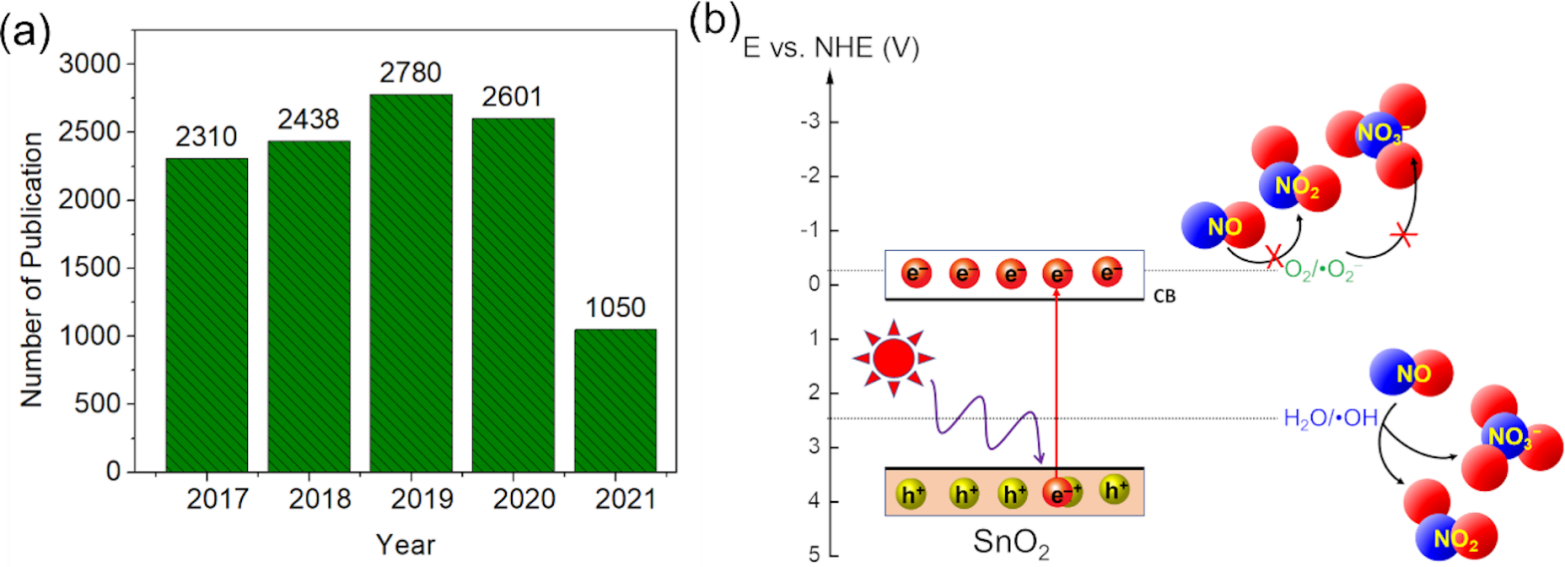
(a) Statistics of publication number on SnO_2_ materials (2017–06/2021). Data was extracted from Web of Science, Clarivate Analytics; (b) NO photocatalytic oxidation ability of SnO_2_.

Recently, research on tin dioxide (SnO_2_) materials has increased significantly, which expresses the potential of SnO_2_ materials for the scientific community (Figure 2a). SnO_2_ is one of the most extensively investigated n-type semiconductors. It is known as tin(VI) oxide or stannic oxide (not to be confused with stannous oxide with tin in the oxidation state of 2+ [13], also known as cassiterite [14]. SnO_2_ materials have many interesting properties. For instance, the structure and electronic structure can be manipulated easily due to the highly tunable valence state and oxygen vacancy defects (OVs) [15–16]. Therefore, SnO_2_ is considered a potential material in various technological fields such as catalysis, optoelectronic devices, rechargeable lithium batteries, electrocatalysis, photocatalysis, solar energy conversion, and gas sensing [17–24]. In the catalytic area, SnO_2_ is an emerging material for removing contaminants such as organic dyes, phenolic compounds, and volatile organic compounds (VOCs) due to strongly oxidizing properties thanks to flexible energy band structure, rich defects, good chemical, and high thermal stability, and easily controlled morphology [25–30]. However, pure SnO_2_ suffers from some inherent drawbacks that limit its practical applications. With a wide bandgap (3.5–3.7 eV) [31–32], SnO_2_ can only be excited by UV irradiation. As a typical oxidation photocatalyst with the CB edge energy level, which is not conducive to the reduction of O_2_ to ^•^O_2_^−^ööö[31,33] and the rapid recombination rate of photoinduced electron–hole pairs [34], the photocatalytic ability of SnO_2_ is less efficient than that of other semiconductor photocatalysts (Figure 2b). Despite literature relating to the unfavorable CB edge of SnO_2_, many reports still proposed its photocatalytic behaviors partly based on ^•^O_2_^−^ species via the combination of experimental physicochemical analyses, such as electron spin resonance (ESR) spectroscopy, active species trapping experiments, valence band X-ray photoelectron spectroscopy (XPS), and diffuse reflectance spectroscopy (DRS) [35–40]. This promotes a new avenue for diverse analyses of semiconductor photocatalysts in addition to the traditional theories and conclusions.

Previous studies have shown that the photocatalytic activity of NO*_x_* decomposition of materials in general and SnO_2_ depends on many factors, including the structure and energy band, surface and defect states, morphology, etc. For that reason, recent studies are being focused on the modification of properties of SnO_2_ to upgrade the photocatalytic efficiency of SnO_2_, including bandgap engineering, defect regulation, surface engineering, heterojunction construction, co-catalyst, which will be thoroughly outlined in this review.

### Structure and bandgap

SnO_2_ has a crystal structure similar to that of rutile TiO_2_ööö[41–42]. The unit cell parameters of rutile SnO_2_ are *a* = *b* = 0.47374 nm and *c* = 0.31864 nm [43]. In one unit cell of rutile SnO_2_, a Sn^4+^ ion is bonded to six oxygen ions, and every oxygen atom is coordinated by three Sn^4+^ ions, forming a (6, 3) coordination structure [44]. When SnO_2_ materials are prepared as thin films with two to eight layers the bandgap is larger than that of bulk SnO_2_ and decreases with increasing film thickness [45]. Zhou et al. indicated that the direct bandgap transition of SnO_2_ has an absorption coefficient α and the optical bandgap (*E*_g_) can be determined by the calculation of *α*(*h*ν)^2^ ∝ (*h*ν − *E*_g_)^1/2^/*h*ν*,* and the plot of *α*(*h*ν)^2^ vs photon energy *h*ν, respectively. For example, the bandgap of a SnO_2_ thin film with a thickness of about 130 nm is 3.597 eV [42].

**Figure 3:**
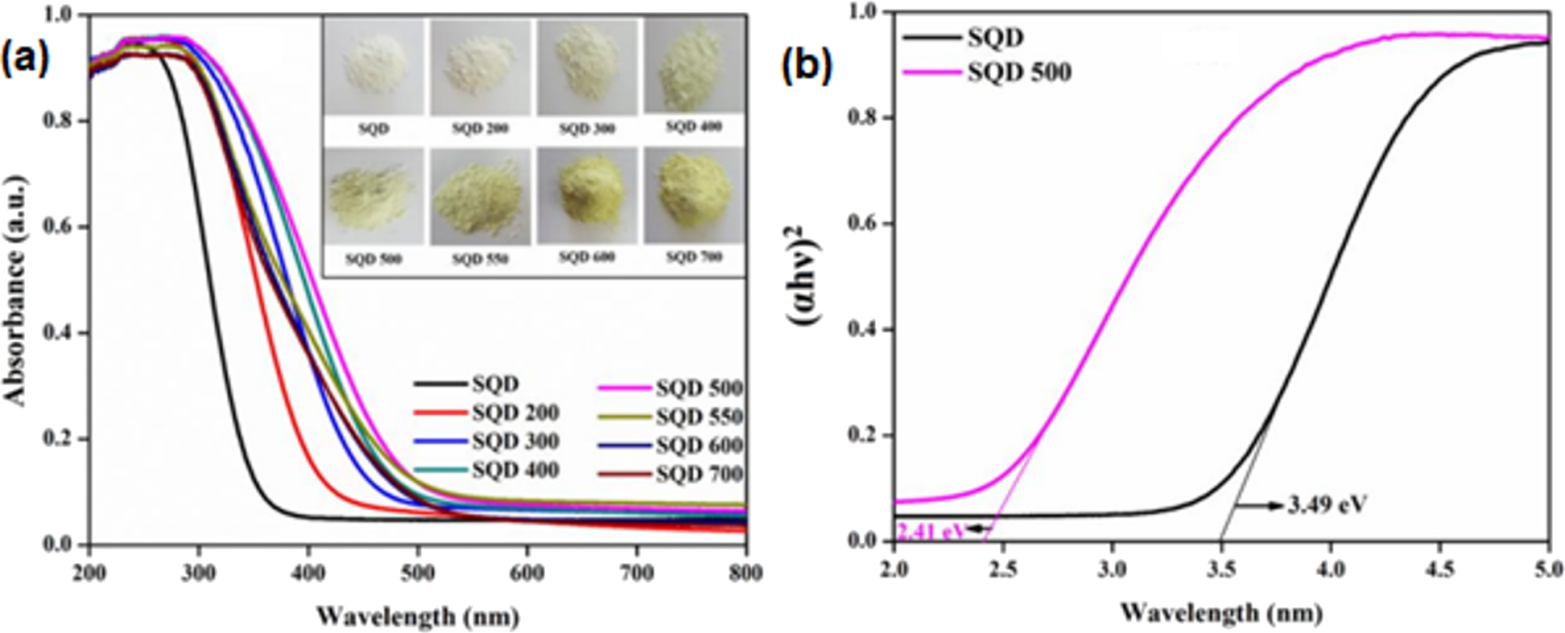
Ultraviolet–visible absorption spectra (a) and corresponding bandgaps of SQDs (b). Figure 3 was reprinted from [46], Materials Letters, vol. 221, by Babu, B.; Neelakanta Reddy, I.; Yoo, K.; Kim, D.; Shim, J. “Bandgap tuning and XPS study of SnO_2_ quantum dots”, pages 211–215, Copyright (2018), with permission from Elsevier. This content is not subject to CC BY 4.0.

The reported bandgap of bulk SnO_2_ is 3.6 eV. Changing the morphology, particle size, or the formation of OVs or defects narrow the bandgap. In the study of Babu et al., a redshift of the absorption edge was observed when SnO_2_ quantum dots (SQDs) were heated from 200 to 700 °C, which indicated that the bandgap of the SQDs decreased from 3.49 to 2.52 eV (for SQD-700) as shown in Figure 3. These results demonstrated that the redshift is favorable for a photocatalytic activity in the visible light region.

Meanwhile, Fan et al. [47] investigated the bandgap of SnO_2_ when changing the self-doping of SnO_2_. The change of the color of the powder products and the redshift in the absorption spectra are two quantities that are correlated with each other. Normally, SnO_2_ is white and optical absorptions in the visible region arise from changes of the band structure. Moreover, the bandgap of SnO_2−_*_x_* self-doped with Sn^2+^ can be easily determined as follows: A straight line to the *x*-axis, equaling to the extrapolated value of *E*_photon_ at α = 0, gives the absorption edge energy. This energy parameter corresponds to the bandgap (*E*_g_) of the material [47].

### Surface and defect states

**Figure 4:**
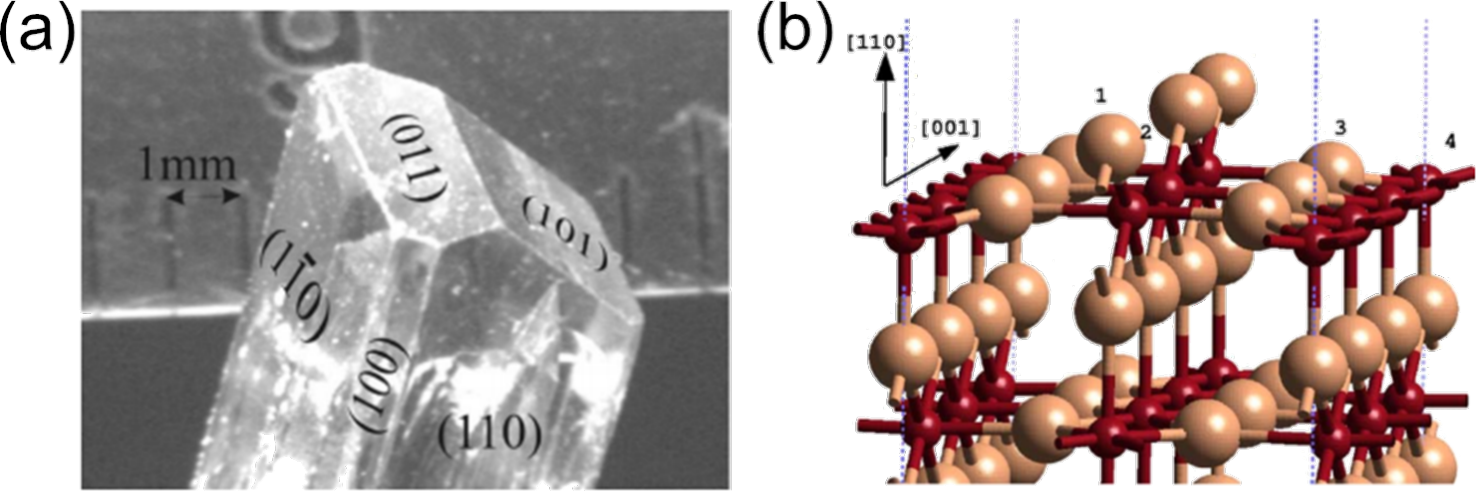
(a) Natural growth faces of SnO_2_ are the (110), (100) (equivalent to (010) in rutile), and (101) (equivalent to (011) in rutile) surfaces. Figure 4a was reprinted with permission from [48] (M. Batzill; K. Katsiev; J. M. Burst; U. Diebold; A. M. Chaka; B. Delley, Phys. Rev. B, vol. 72, article no. 165414, 2005). Copyright (2005) by the American Physical Society. This content is not subject to CC BY 4.0; (b) SnO_2_(110) surface including a bridging oxygen vacancy (1-bridging oxygen; 2-bridging OV; 3-oxygen coordinated threefold with surface tin (Sn 5f); 4-oxygen coordinated fivefold with surface tin (Sn 5f). Figure 4b was reprinted from [53], Surface Science, vol. 577, by Mäki-Jaskari, M. A.; Rantala, T. T.; Golovanov, V. V. “Computational study of charge accumulation at SnO_2_(110) surface”, pages 127–138, Copyright (2005), with permission from Elsevier. This content is not subject to CC BY 4.0.

Structural defects and lattice imperfections usually bestow most of the properties exploited for applications of SnO_2_ materials as they influence various physicochemical properties and reactions on the surface. Most important are defect states of materials, including predominantly point defects, that is, defects associated with one lattice point, such as cation or oxygen ion vacancies. OVs determine the physical and chemical properties of metal oxides. Figure 4a shows the natural crystal structure of SnO_2_ synthesized by vapor transport [48]. The (110) plane of rutile SnO_2_ is the most common surface, and it is also thermodynamically the most stable [48]. In the rutile phase of SnO_2_ in Figure 4b, the (110) plane contains all surface bridging oxygens (1), bridging OVs (2), and oxygen coordinated three- or fivefold (3, 4) with surface tin atoms (Sn 5f). The dual valency of Sn at the surface of SnO_2_ plays a role in the reversible transformation of the surface composition from Sn^4+^ cations to Sn^2+^, which leads to active centers in the surface chemical process [48]. Moreover, the OVs in SnO_2_ often appear when it is synthesized by chemical methods such as sol–gel, hydrothermal, and microwave synthesis [49–51]. The formation and concentration of OVs depend on particle size, synthesizing temperature, and morphology of SnO_2_. The OVs play the role of an electron donor and provide free electrons, making SnO_2_ an n-type semiconductor [52].

**Figure 5:**
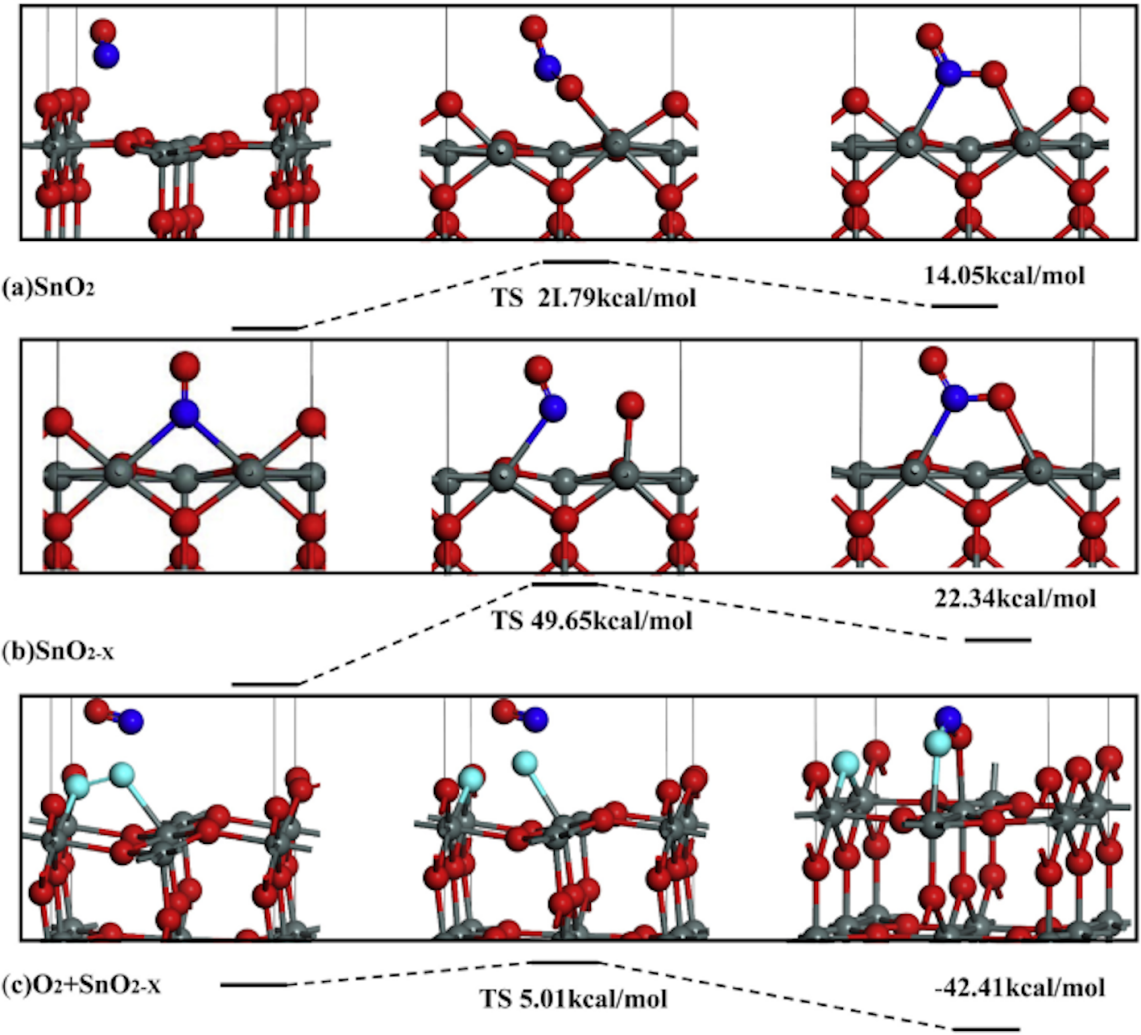
The conversion processes of NO on perfect SnO_2_(110), SnO_2−_*_x_*(110) and O_2_ + SnO_2−_*_x_*(110) surfaces. Figure 5 was reprinted from [54], Sensors and Actuators B: Chemical, vol. 221, by Xu, G.; Zhang, L.; He, C.; Ma, D.; Lu, Z. “Adsorption and oxidation of NO on various SnO_2_(110) surfaces: A density functional theory study”, pages 717–722, Copyright (2015), with permission from Elsevier. This content is not subject to CC BY 4.0.

Guoliang Xu et al. indicated that NO could be absorbed easily on various SnO_2_(110) surfaces, and it is preferentially adsorbed on the OV site through an N-down orientation. Figure 5 shows the calculation of the energy of NO conversion processes on SnO_2_(110), SnO_2−_*_x_*(110), and O_2_ + SnO_2−_*_x_*(110) surfaces. The oxidation of NO on other surfaces is determined by the reaction energies, as shown in Figure 5. The O_2_ + SnO_2−_*_x_*(110) surface is more exothermic and preferable than other surfaces, which leads to an efficient reaction of NO with the SnO_2_ surface [54]. Also, Tiya-Djowe et al. [55] indicated that calcined SnO_2_ samples with higher OV density showed improved photocatalytic performances. Besides, the OV density contributes to the rise of the valence band maximum and a decrease of the bandgap energy of SnO_2_ materials.

### Morphology

There are many shapes of SnO_2_, for example, nanoparticles, nanocubes, nanorods, nanosheets, nanospheres, nanobelts, and nanotubes. These morphologies can be controllably obtained by using polyvinylpyrrolidone (PVP), sodium dodecyl sulfonate (SDS), cetyl trimethyl ammonium bromide (CTAB), or tetrapropyl ammonium bromide (TPAB) as surfactants in a hydrothermal method [56–59]. The difference of morphologies will affect the properties of SnO_2_ regarding gas sensor activity and optical, electrical, and electrochemical properties [60–63]. The typical properties of SnO_2_ are significantly affected by the effective surface area of different nanomaterial morphologies [63–65].

**Figure 6:**
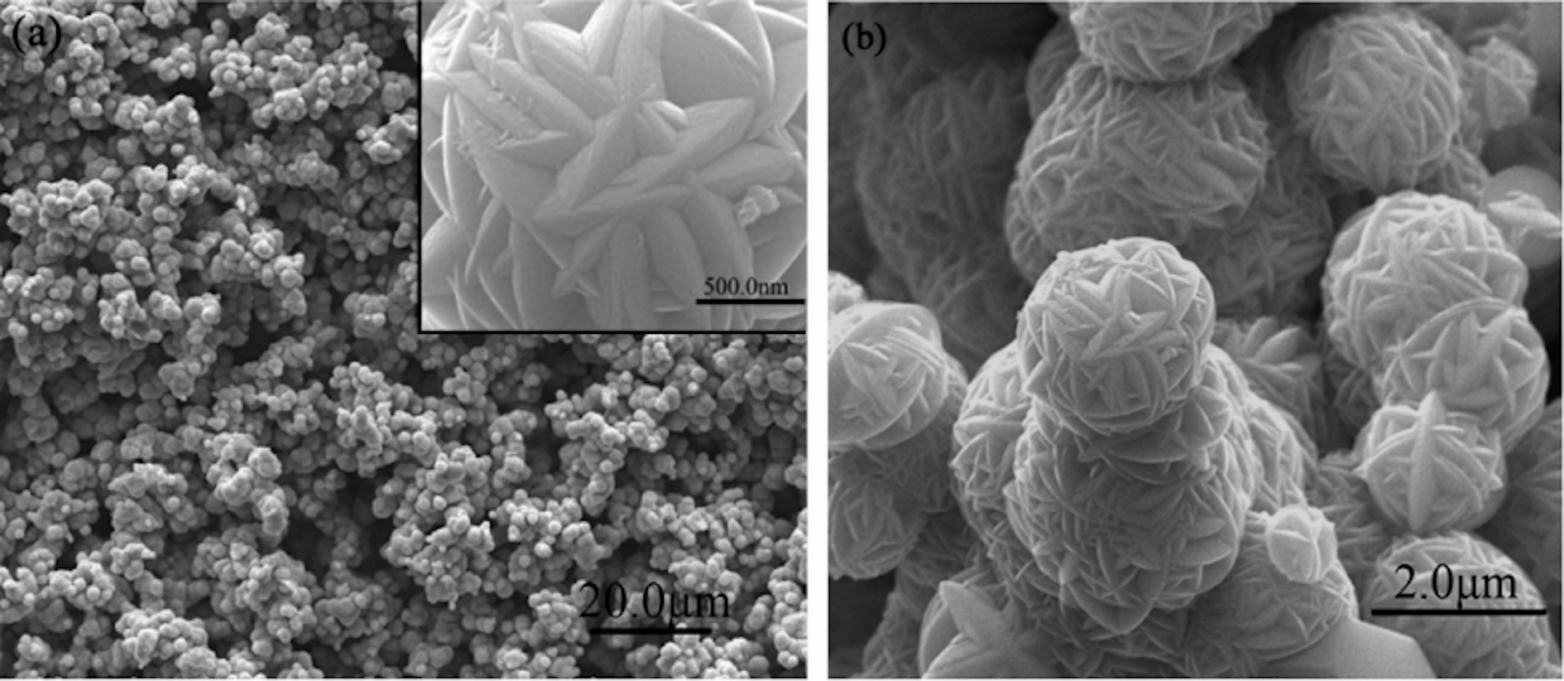
SEM images of SnO_2_ microspheres synthesized by a hydrothermal method at 180 °C for 24 h. Figure 6 was reprinted with permission from [66], Copyright 2010 American Chemical Society. This content is not subject to CC BY 4.0.

**Figure 7:**
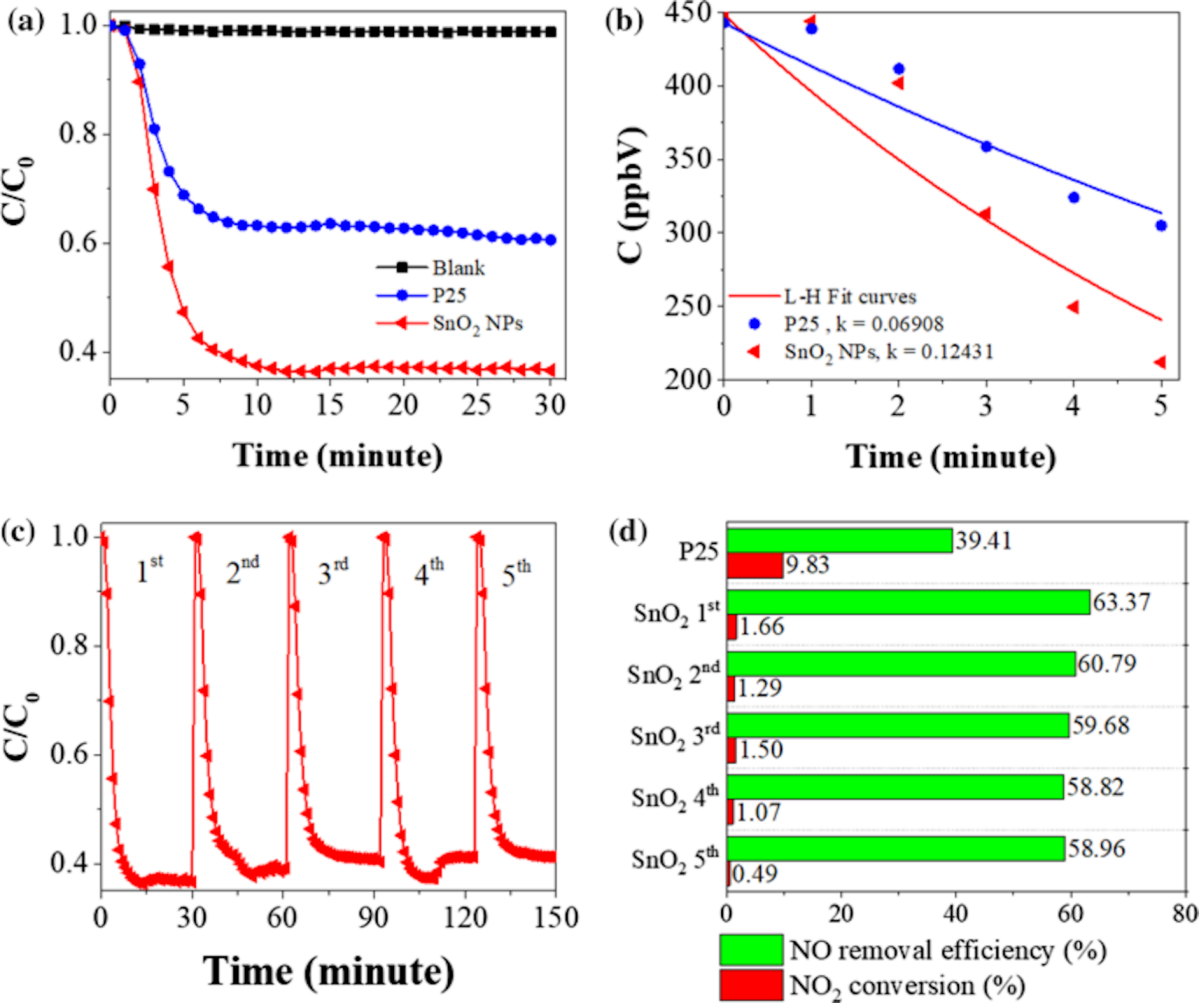
NO photodegradation of materials under solar light (a), the dependence of concentration on irradiation time (b), photochemical stability of SnO_2_ NPs (NPs) (c), and NO removal efficacy and NO_2_ conversion efficacy (d). Figure 7 is from [69] and was reprinted by permission from Springer Nature from the journal Environmental Chemistry Letters (“High photocatalytic removal of NO gas over SnO_2_ nanoparticles under solar light” by T. H. Huy; B. D. Phat; C. M. Thi; P. V. Viet), Copyright 2018 Springer Nature. This content is not subject to CC BY 4.0.

Wang et al. [66] synthesized SnO_2_ microspheres on a fluorine-doped tin oxide (FTO) substrate and the SEM images (Figure 6) show SnO_2_ microspheres with an average diameter of 2.0–2.5 μm. By using SnO_2_ microsphere photocatalysts for the photocatalytic oxidation of NO, Le et al. [67] indicated that 3D hierarchical flower-like SnO_2_ microspheres exhibited a photocatalytic activity towards NO decomposition comparable to that of commercial P25 TiO_2_. Specifically, SnO_2_ microspheres can degrade 57.2% NO (1 ppm of initial concentration) under solar light. However, the photocatalytic mechanism of NO degradation has not been investigated [67]. Zhang et al. [68] found that the crystalline/amorphous stacking structure of SnO_2_ microspheres can moderate surface absorption competition between oxygen gas and NO gas, contributing to the generation of reactive oxygen species (ROS) to oxidize NO to NO_3_^−^ ions. Huy et al. [69] synthesized SnO_2_ NPs, and this is the first report on using a SnO_2_ photocatalyst with NP morphology for the NO degradation. The photocatalytic mechanism of SnO_2_ NPs is based on electrons and holes to generate reactive radicals. Figure 7 shows that the photocatalytic NO removal efficacy of SnO_2_ NPs achieved 63.37% after 30 min under solar light irradiation, and the conversion efficacy from NO to NO_2_ is 1.66%. The high photocatalytic performance and the stability of SnO_2_ NPs under solar light is promising for potential application [69].

### Recent approaches in the modification of SnO_2_ for photocatalytic NO*_x_* oxidation

Many attempts have been made to enhance the photocatalytic activity and take better advantage of SnO_2_ for the NO*_x_* abatement, including the combination with other metal oxides [70], organic semiconductors [71], or metallic nanomaterials [72] to form a heterojunction/composite photocatalyst, and self-doping [73] or elemental doping [39,74]. Hybrid or doped photocatalysts ideally exhibit an improved photocatalytic efficacy due to the reduced recombination rate of photogenerated charge carriers and the lower activation energy. However, additional factors considerably affect the overall photocatalytic process. Table 1 shows a comparison of the NO photocatalytic oxidation ability of neat SnO_2_ and modified SnO_2_ materials. Recent studies on this material system mainly focus on modifying SnO_2_ toward the application in the visible light region.

**Table 1:** A comparison of photocatalytic systems for NO abatement with SnO_2_ photocatalyst systems.

Year	Photocatalyst	SnO_2_ morphology	Experimental conditions				NO removal (%)	NO_2_ yield (%)	Ref.
			
			Light source	Initial NO conc. (ppb)	Humidity (%)	Sample weight (g)			

2013	SnO_2_	microspheres	vis: λ > 510 nm and λ > 400 nm; UV: λ > 290 nm (450 W high-pressure mercury lamp with filters)	10^3^	N/A	N/A	57.2 (λ > 290 nm) 11.5 (λ > 400 nm) 4.2 (λ > 510 nm)	N/A	[67]
2017	SnO_2_/Zn_2_SnO_4_/graphene	unclear shape	vis (3 W LED lamp, λ = 420 ± 10 nm)	600	N/A	0.2	59.3	N/A	[75]
2018	SnO_2_	NPs	solar (300 W Xe lamp)	450	70	0.2	63.37	1.66	[69]
2018	SnO_2_/TiO_2_	NPs	vis (300 W Xe lamp with a UV cutoff filter (λ > 420 nm)	450	70	0.2	59.49	2.58	[38]
2018	SnO_2_/graphene	QDs	solar and vis (Xe lamp)	600	N/A	N/A	75 (full spectrum) 57 (vis)	N/A	[36]
2018	SnO_2_/polyaniline	NPs	solar (300 W Xe lamp)	450	30	0.2	15	8	[35]
2019	SnO_2_/N-doped carbon quantum dots/ZnSn(OH)_6_	NPs	vis-near-infrared (300 W Xe lamp, λ ≥ 420 nm)	400	30 ± 5	0.2	37	<1.25	[76]
2019	SnO_2_/g-C_3_N_4_	QDs	vis (150 W tungsten halogen lamp with a filter (λ > 420 nm)	600	N/A	0.4	32	8	[37]
2019	Ag@SnO_2_	NPs	solar (300 W Xe lamp)	N/A	N/A	0.2	70	4	[72]
2020	Ce doped SnO_2_	particles	vis (300 W Xe lamp with a UV filter (λ > 420 nm)	10^4^	65	0.4	82	10	[39]
2020	BiOBr/SnO_2_	NPs	vis (150 W tungsten halogen lamp with a UV cut-off filter (λ > 420 nm)	600	N/A	0.10	50.3	N/A (NO-to-NO_2_ conversion was studied via in situ DRIFTS)	[70]
2021	g-C_3_N_4_/SnO_2_	NPs	vis (300 W solar simulator with a UV filter (λ > 420 nm)	500	70	0.2	44.17	9.29	[71]
2021	SnO_2−_*_x_*/g-C_3_N_4_	NPs	vis (300 W solar simulator with a UV cut-off filter (λ > 420 nm)	500	N/A	0.2	40.8	7.5	[73]

### Charge transfer improvement

The combination of SnO_2_ with other co-photocatalysts, including inorganic and organic semiconductors, is a practical approach to enhance the charge transfer efficacy for the photocatalytic process. The photocatalytic degradation of NO*_x_* over SnO_2_ as a host photocatalyst is reported to be considerably enhanced after the combination with organic semiconductors such as graphitic carbon nitride (g-C_3_N_4_) [71]. When acting as an auxiliary photocatalyst, SnO_2_ promotes the photocatalytic activity of the primary material [38,70,75–76].

**Figure 8:**
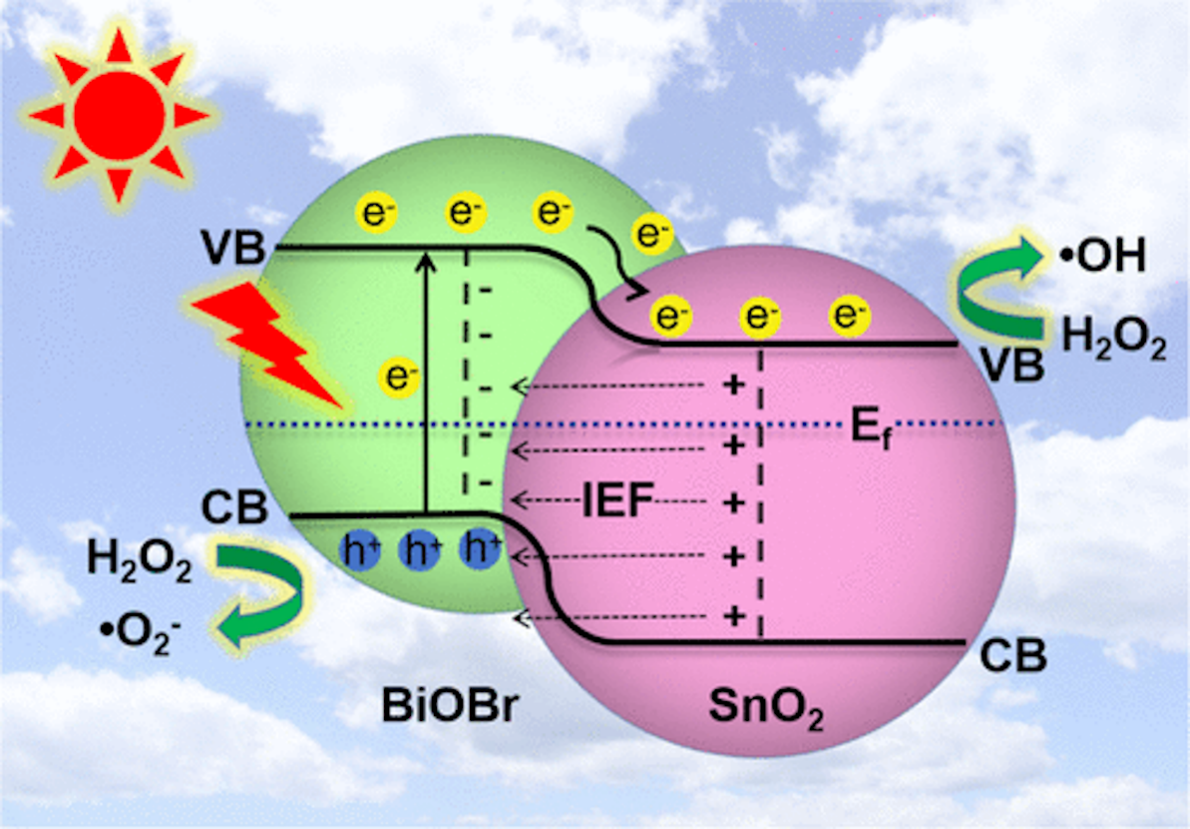
Proposed mechanisms for photocatalytic NO oxidation via interfacial charge migration over BiOBr/SnO_2_ p–n heterojunctions. Figure 8 was reprinted with permission from [70], Copyright 2020 American Chemical Society. This content is not subject to CC BY 4.0.

Wu et al. reported the visible-light-driven elimination of NO over hydrothermally synthesized BiOBr/SnO_2_ p–n heterojunction photocatalysts. The as-prepared BiOBr/SnO_2_ photocatalayst with a molar ratio of 2:5 between SnO_2_ NPs and BiOBr microspheres shows an enhanced NO*_x_* photocatalytic removal of 50.3%, at an initial NO concentration of 600 ppb, and a great stability after four cycles. The generation of toxic NO_2_ products was inhibited effectively. The charge movement at the BiOBr/SnO_2_ p–n interface was also revealed via theoretical and experimental findings. Electrons in SnO_2_ transfer into BiOBr over pre-formed charge migration channels and an internal electric field at the BiOBr/SnO_2_ interface, which directs photoinduced electrons from the CB of BiOBr to that of SnO_2_, thus prolonging the lifetime of photogenerated electron–hole pairs (Figure 8). The NO-to-NO_2_ conversion and intermediates and products were confirmed via in situ diffuse reflectance infrared Fourier transform spectroscopy during NO oxidation [70].

**Figure 9:**
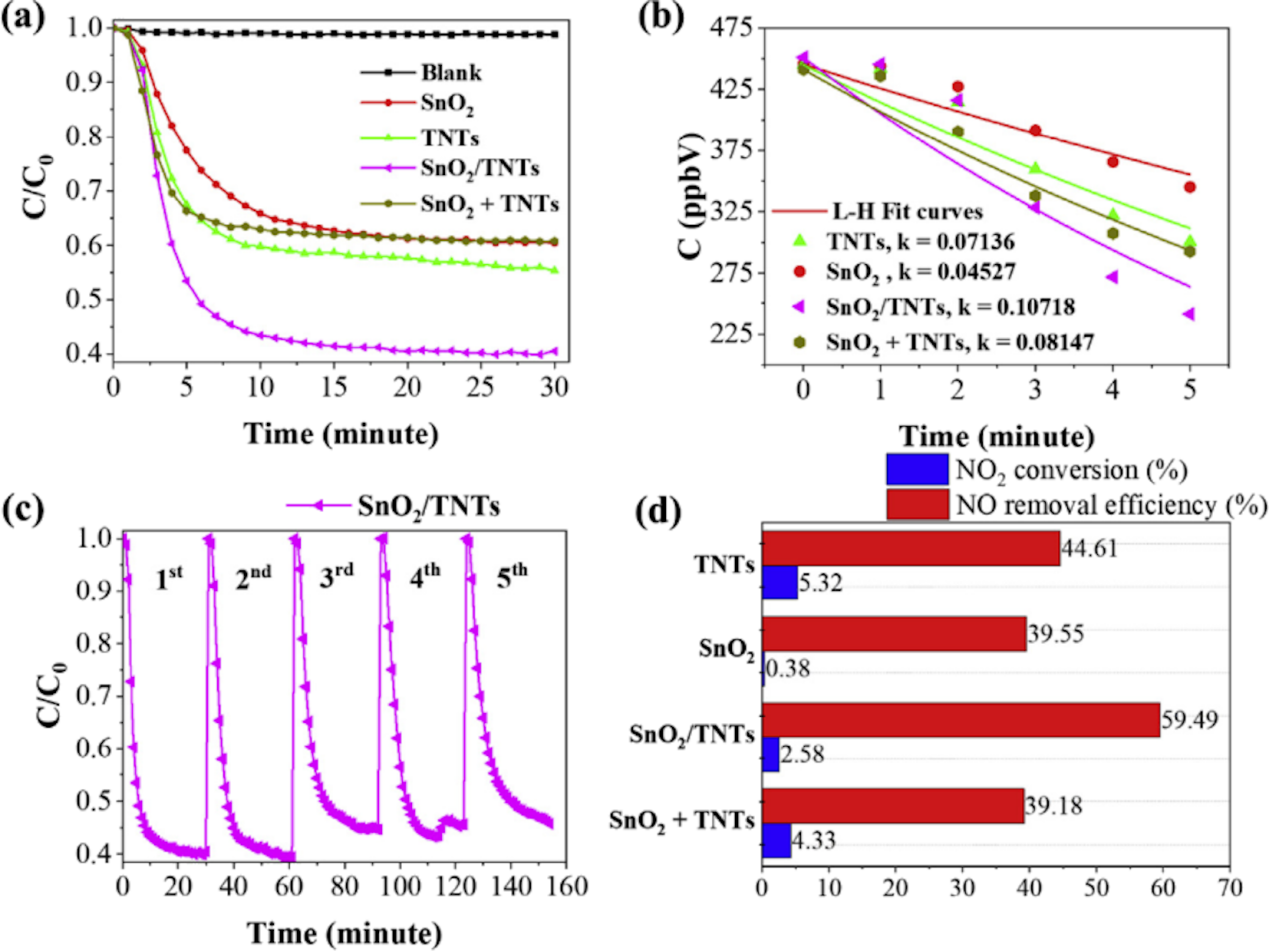
NO photocatalytic degradation of materials under visible light irradiation (a), the dependence of concentration on irradiation time (b), photochemical stability of SnO_2_/TNTs (c), and NO removal efficacy and NO_2_ conversion efficacy (d). Figure 9 was reprinted from [38], Chemosphere, vol. 215, by Huy, T. H.; Bui, D. P.; Kang, F.; Wang, Y. F.; Liu, S. H.; Thi, C. M.; You, S. J.; Chang, G. M.; Pham, V. V. “SnO_2_/TiO_2_ nanotube heterojunction: The first investigation of NO degradation by visible-light-driven photocatalysis”, pages 323–332, Copyright (2018), with permission from Elsevier. This content is not subject to CC BY 4.0.

Huy et al. [38] hydrothermally synthesized SnO_2_ NPs adhering to TiO_2_ nanotubes (SnO_2_/TNTs) via a facile one-step method for the photocatalytic abatement of NO under visible light (Figure 9)*.* At a NO concentration of 450 ppb in a continuous flow, SnO_2_/TNTs yields a photocatalytic degradation of NO of 59.49%, which is much better than that of bare TiO_2_ NTs (44.61%), SnO_2_ NPs (39.55%), and a physical blend of SnO_2_ NPs and TiO_2_ NTs (39.18%). Also, the heterostructured photocatalyst shows an effective reduction of NO_2_ generation after 30 min of photocatalytic reaction. The photogenerated electrons and ^•^O_2_^−^ radicals played a primary role in the photocatalytic NO oxidation. Additionally, using photoluminescence (PL) spectroscopy, XPS, active species trapping tests, and ESR spectroscopy, the authors studied the photoinduced charge migration and trapping. They proposed the band structure of the SnO_2_/TNTs and pointed out the existence of ^•^O_2_^−^ and ^•^OH radicals as critical factors in the photocatalysis process [38]. These results demonstrated that the SnO_2_ NPs could be both a host or an auxiliary material for the NO photocatalytic degradation.

Besides the coupling with semiconductor oxides such as TiO_2_ and BiOBr, recent works reported the successful combination of SnO_2_ nanomaterials with conjugated polymers such as graphitic carbon nitride (g-C_3_N_4_) and polyaniline (PANI), yielding metal-free visible-light-driven photocatalysts for addressing NO gas pollution. Such combinations hold great potential because they exhibit a wide range of useful properties, including high conductivity, cost-effectiveness, high flexibility and processability, and ease of fabrication. These recent advances are highlighted and discussed in terms of preparation method and photocatalytic mechanism in this review. Regarding g-C_3_N_4_, Zou et al. successfully deposited SnO_2_ quantum dots (QDs) on g-C_3_N_4_ sheets by a simple physical mixing process. The authors indicated that the SnO_2_/g-C_3_N_4_ photocatalyst had a twice as high NO removal efficacy than bare SnO_2_ QDs and a low NO_2_ generation upon exposure to visible light for 30 min. This enhancement of the photocatalytic activity was interpreted as the synergistic effect between the high photo-oxidation ability of SnO_2_ triggered by the visible light response of g-C_3_N_4_. Also, the key role of the SnO_2_/g-C_3_N_4_ interface in inhibiting the production of NO_2_ facilitates the transition of photogenerated carriers used for the NO removal [37].

**Figure 10:**
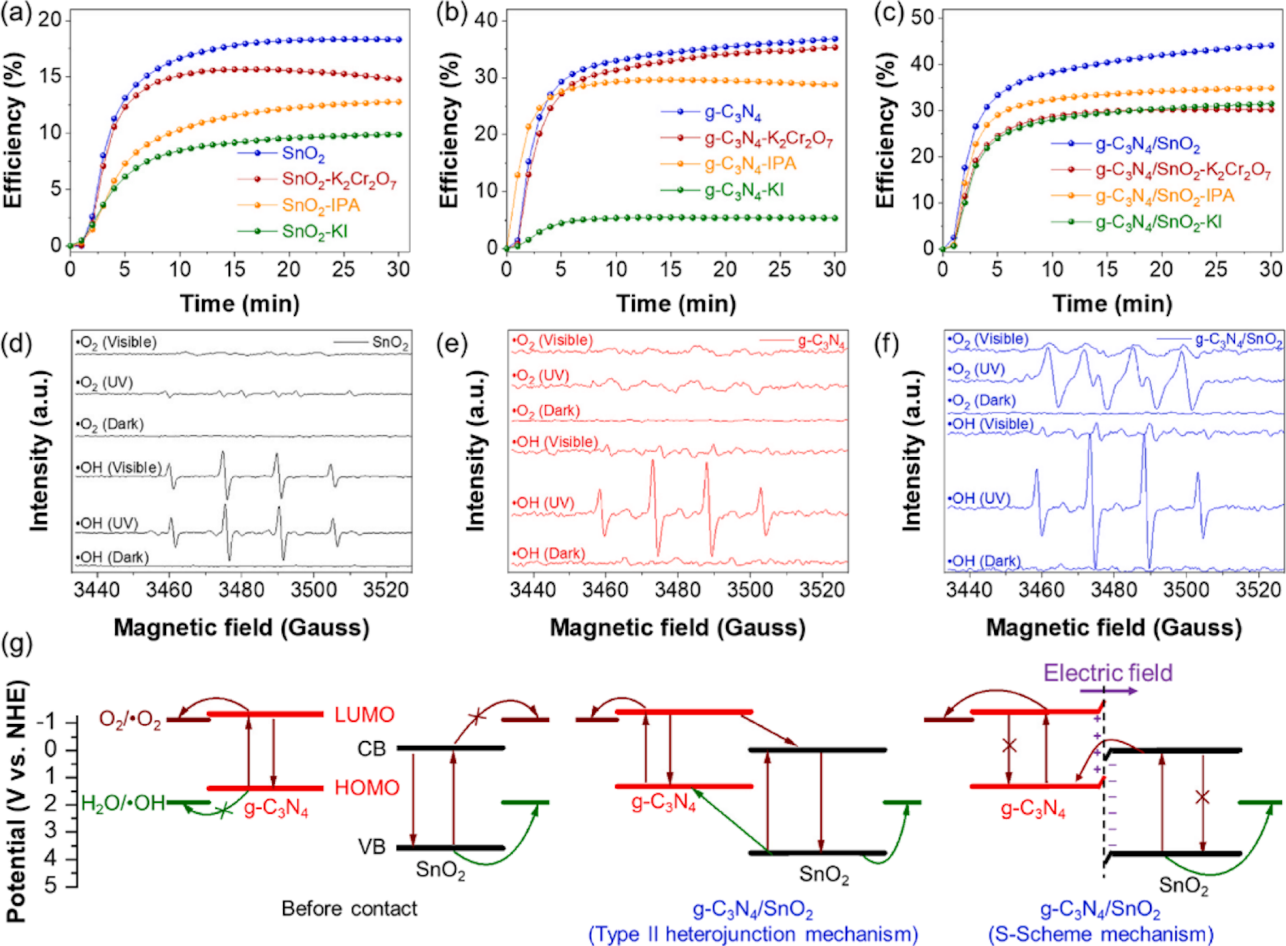
Photocatalytic NO removal efficacy over SnO_2_ (a), g-C_3_N_4_ (b) and g-C_3_N_4_/SnO_2_ (c) with scavengers under visible light (400 *<* λ *<* 800). ESR signals (d) of ^•^OH radicals, and ^•^O_2_^−^ radicals of the materials after 10 min under visible light (400 *<* λ *<* 800). Growth curves of ^•^OH radicals (e) and ^•^O_2_^−^ radicals (f) vs irradiation time of the materials. The charge transfer pathways of the materials (g). K_2_Cr_2_O_7_, KI, and isopropyl alcohol (IPA) act as scavengers for electrons, holes, and ^•^OH radicals, respectively. The brown and green arrows indicate the path of electrons and holes, respectively. Figure 10 was reprinted from [71], Environmental Pollution, vol. 286, by Van Pham, V.; Mai, D.-Q.; Bui, D.-P.; Van Man, T.; Zhu, B.; Zhang, L.; Sangkaworn, J.; Tantirungrotechai, J.; Reutrakul, V.; Cao, T. M. “Emerging 2D/0D g-C3N4/SnO2 S-scheme photocatalyst: New generation architectural structure of heterojunctions toward visible-light-driven NO degradation”, article no. 117510, Copyright (2021), with permission from Elsevier. This content is not subject to CC BY 4.0.

Pham et al. showcased a step-scheme (S-scheme) photocatalyst composed of 2D/0D g-C_3_N_4_ nanosheet-assisted SnO_2_ NPs (g-C_3_N_4_/SnO_2_) for removing NO with low NO_2_ generation. This work established an S-scheme charge transfer path by combining density functional theory (DFT) calculations, trapping experiments, and electron spin resonance measurements (Figure 10). Thus, the impact of intrinsic OVs within SnO_2_ NPs and the resulting S-scheme heterojunction on the band structure, charge transfer, and photocatalytic activity was presented. The resulting heterojunction photocatalytically removed 40% NO (initial concentration of 500 ppb) and showed excellent photostability under visible light. The NO_2_ production from the photocatalytic reaction was also negligible. The good photocatalytic NO degradation of the 2D/0D g-C_3_N_4_/SnO_2_ catalyst is due to the defects actively trapping electrons and the charge transfer described in the S-scheme model. These factors increase the lifetime of electron–hole pairs and free radicals. The finding of this work enables the generation of a new and innovative structures with S-scheme heterojunctions for environmental treatment [71].

**Figure 11:**
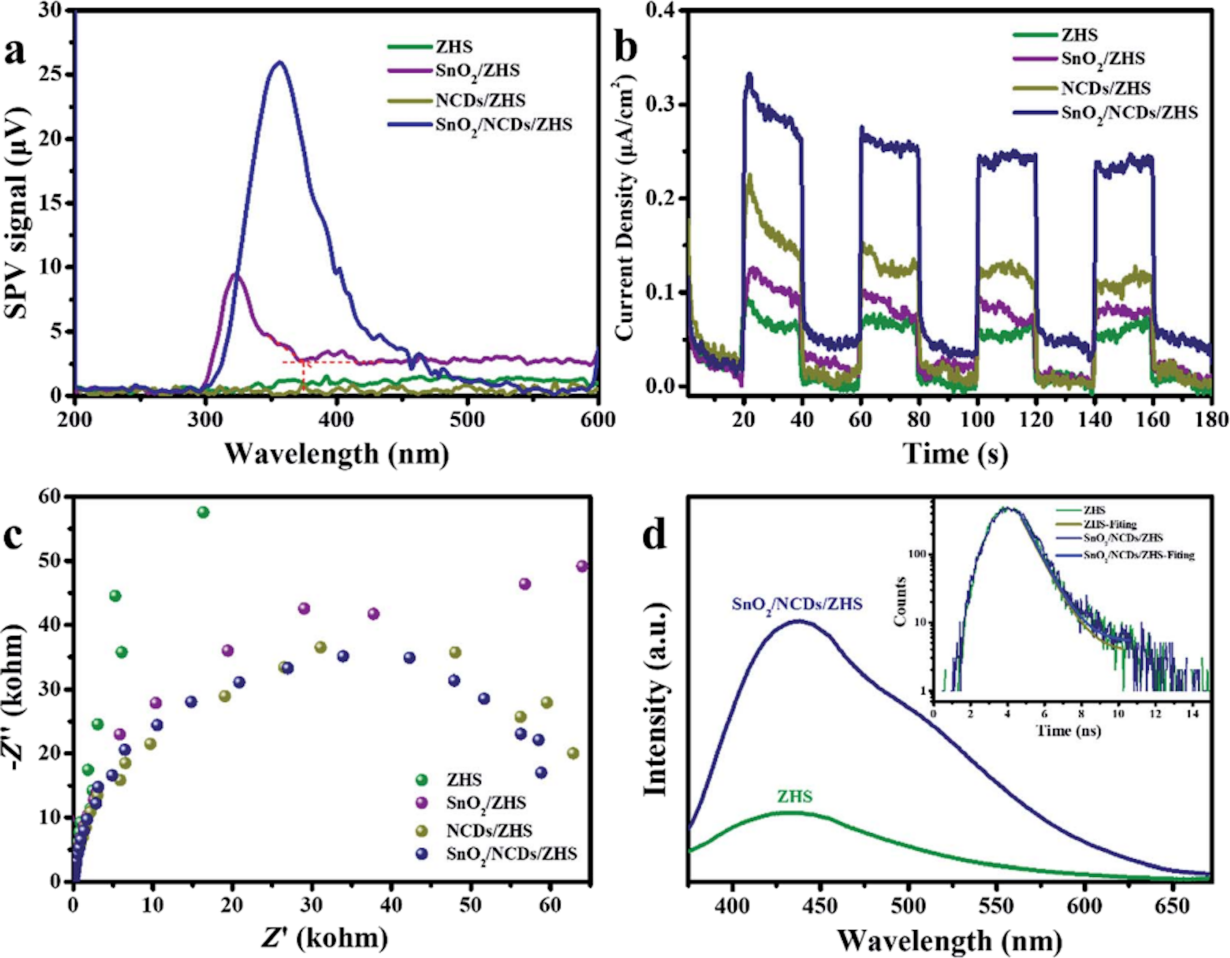
(a) Surface photovoltage spectroscopy, (b) transient photocurrent responses, (c) EIS Nyquist plots of ZHS, SnO_2_/ZHS, NCDs/ZHS and SnO_2_/NCDs/ZHS samples, and (d) PL spectra (inset: transient fluorescence decay spectra). Figure 11 was republished with permission of The Royal Society of Chemistry from [76] (“Constructing Z-scheme SnO_2_/N-doped carbon quantum dots/ZnSn(OH)_6_ nanohybrids with high redox ability for NO*_x_* removal under VIS-NIR light” by Y. Lu et al., J. Mater. Chem. A, vol. 7, issue 26, © 2019); permission conveyed through Copyright Clearance Center, Inc. This content is not subject to CC BY 4.0.

A similar model, a Z-scheme photocatalyst, was reported by Lu et al. who successfully fabricated a ternary nanohybrid consisting of mesoporous SnO_2_, nitrogen-doped carbon quantum dots (NCDs), and ZnSn(OH)_6_ using a simple in situ solvothermal method. This nanohybrid photocatalyst exhibited a broad optical response range and excellent oxidation ability and showed great potential in addressing air pollution. The ternary Z-scheme photocatalyst could remove 37% of NO under visible light and IR without generating NO_2_. In addition, this work also discussed the critical role of NCDs in extending the light harvesting range and promoting the separation of photogenerated electrons. A considerable amount of reactive oxygen radicals was produced during the photocatalytic reaction, resulting from the large amount of free surface OH groups. PL, photocurrent response, electrochemical impedance spectroscopy (EIS) data, and the nanosecond-level time-resolved fluorescence decay spectra (Figure 11) demonstrated that the SnO_2_/NCDs/ZHS nanohybrid achieved low charge carrier recombination, high photoactivity, and excellent photoinduced charge transfer to the surface of the semiconductor. This study enables new insights into the underlying mechanism of heterojunction photocatalysts, especially those with Z-shaped interfaces [76].

Polyaniline (PANI) is a conducting polymer and compared to g-C_3_N_4_, PANI is inexpensive and easy to synthesize. Bui et al. [35] presented a SnO_2_/PANI nanocomposite for photocatalytic NO removal under solar light for the first time. Furthermore, they found that the introduction of SnO_2_ NPs increases the photostability of PANI during the photocatalytic process, which holds great potential for scalable manufacturing. Also, this work thoroughly discussed the adsorption and photocatalytic mechanisms, and the polymer photodegradation of the resulting nanocomposite using DFT techniques. The results confirmed that the interaction between NO and PANI is indeed a hydrogen bond and photogenerated holes serve as the primary factor of the photocatalytic NO removal [35]. Moreover, this study also indicated that hydrogen bonds between NO and PANI increased the adsorption of NO on the SnO_2_/PANI surface, leading to enhanced photocatalysis. However, the photocatalytic stability of SnO_2_/PANI is still a challenging problem.

**Figure 12:**
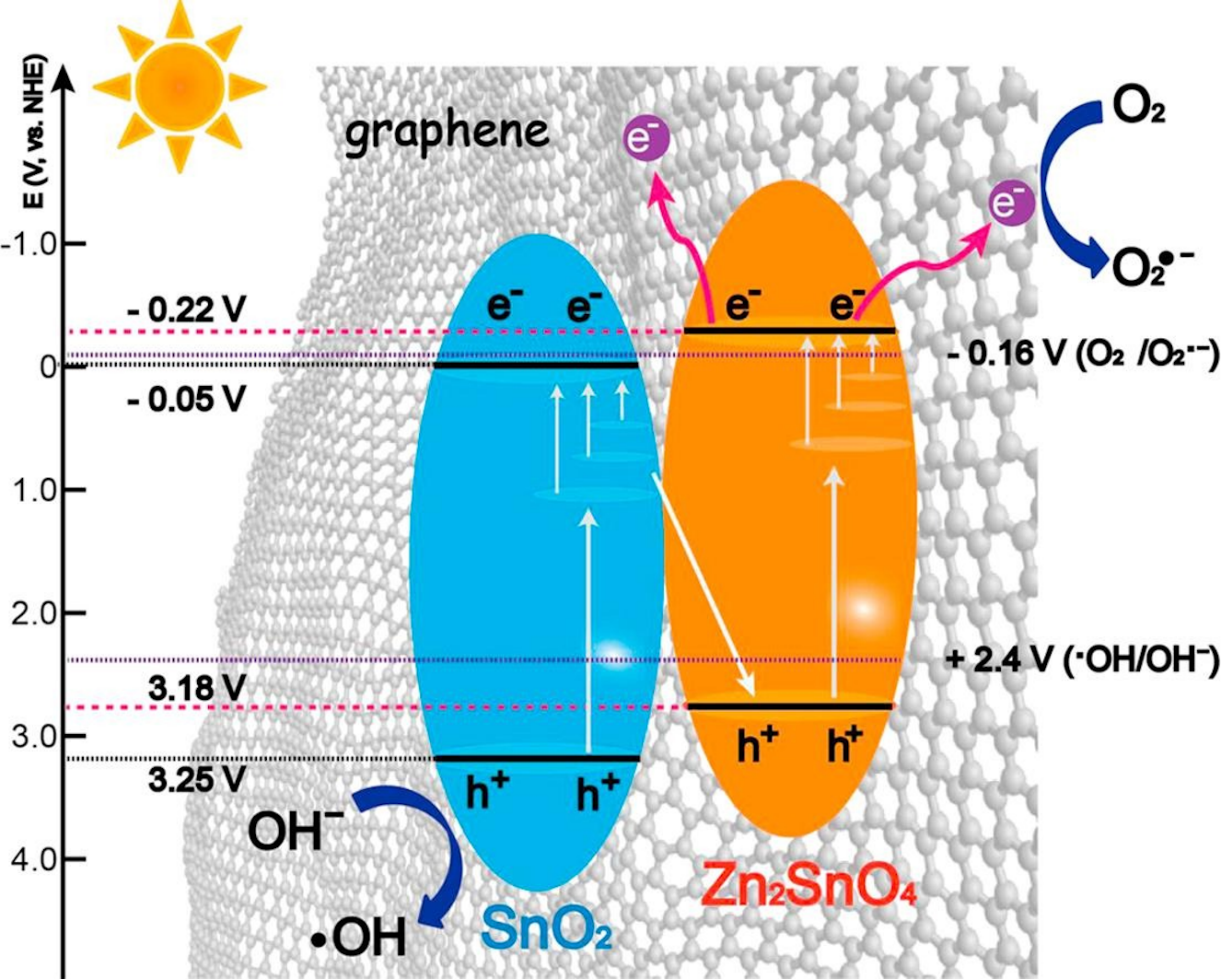
A mechanism of NO photocatalytic oxidation over SnO_2_–Zn_2_SnO_4_/graphene. Figure 12 was reprinted from [75], Chemical Engineering Journal, vol. 336, by Li, Y.; Wu, X.; Ho, W.; Lv, K.; Li, Q.; Li, M.; Lee, S. C. “Graphene-induced formation of visible-light-responsive SnO_2_-Zn_2_SnO_4_ Z-scheme photocatalyst with surface vacancy for the enhanced photoreactivity towards NO and acetone oxidation”, pages 200–210, Copyright (2017), with permission from Elsevier. This content is not subject to CC BY 4.0.

Enesca et al. [29] developed photoactive heterostructures based on SnO_2_, TiO_2_, and CuInS_2_ using an automated spray pyrolysis method, which is particularly beneficial for air cleaning applications. This work showed that the surface tension of the material surface directly impacts the photocatalytic activity under humid conditions. Furthermore, introducing CuInS_2_ enables good UV and vis absorption thus extending the light-responsive range. As a result, such a CuInS_2_/TiO_2_/SnO_2_ heterostructure presented one of the highest photocatalytic efficacies (51.7%) in acetaldehyde removal. However, this work also opens some new questions for future studies on optimizing the band structure, which remains critical for studying charge separation [29]. In another study, a SnO_2_–Zn_2_SnO_4_ Z-scheme photocatalyst system was prepared with a graphene modification to create surface vacancy sites in the composite, which contributed to an enhanced photoactivity in the oxidation of NO and acetone [75]. The presence of graphene induces the formation of SnO_2_ and introduces Sn vacancies, which supports the electron transfer from the CB of Zn_2_SnO_4_ to oxygen under visible light irradiation (Figure 12). The authors only used a visible light LED with low power (3 W) and obtained a high efficacy of NO degradation (59.3%) [75]. However, the disadvantage of this study and other studies is that it did not determine the formation of NO_2_ after the reaction (see Table 1).

### Creation of narrower bandgaps

**Figure 13:**
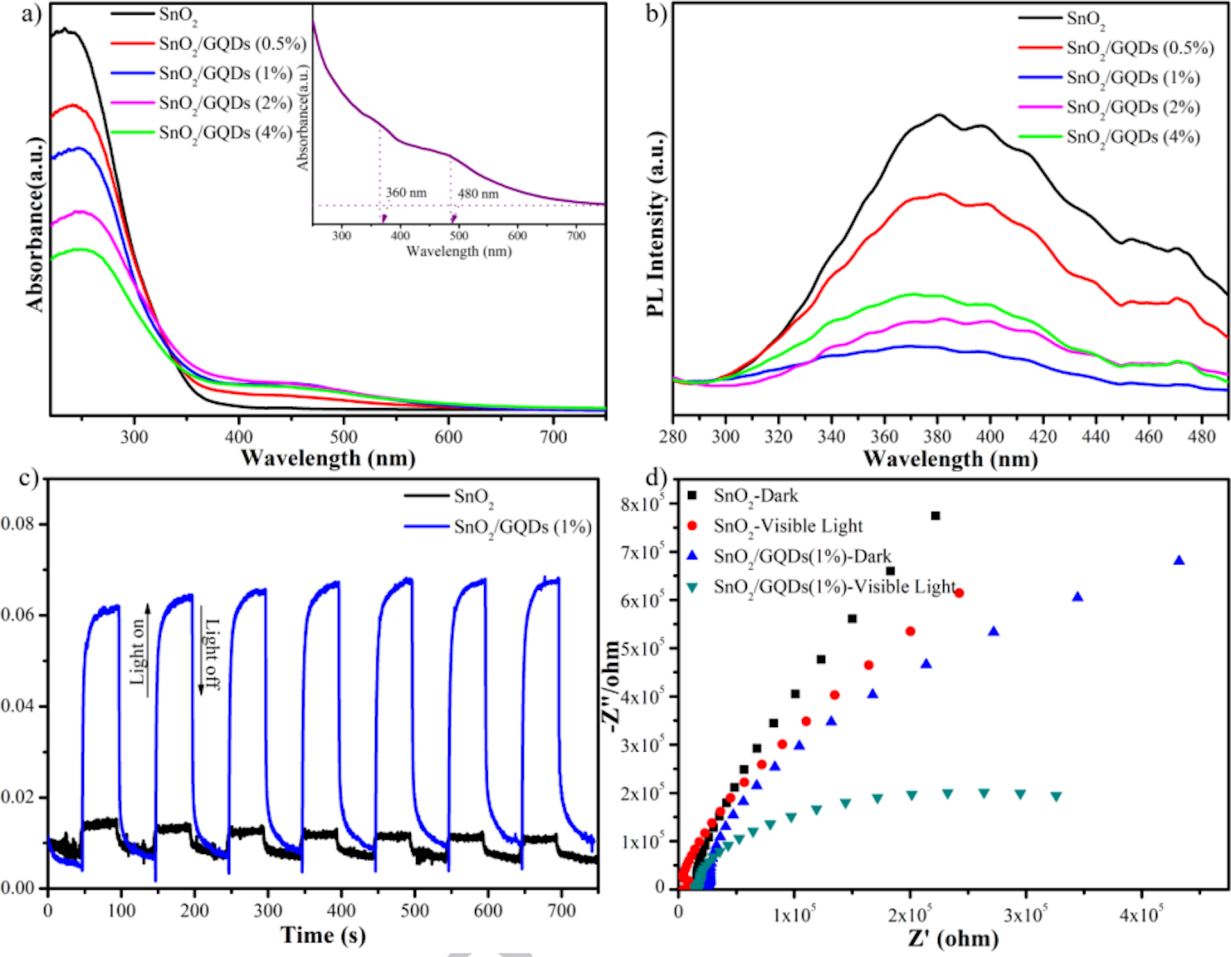
(a) Diffuse reflectance spectra of SnO_2_ and SnO_2_/GQDs composites. Inset is the absorption spectrum of GQDs dispersed in water. (b) PL spectra of SnO_2_ and SnO_2_/GQDs composites. Excitation wavelength: 260 nm. (c) Transient photocurrent response and (d) EIS curves of SnO_2_ and SnO_2_/GQDs (1%) under visible light illumination and in darkness. Figure 13 was reprinted from [36], Applied Surface Science, vol. 448, by Xie, Y.; Yu, S.; Zhong, Y.; Zhang, Q.; Zhou, Y. “SnO_2_/graphene quantum dots composited photocatalyst for efficient nitric oxide oxidation under visible light”, pages 655–661, Copyright (2018), with permission from Elsevier. This content is not subject to CC BY 4.0.

To narrow the bandgap of SnO_2_ is an advanced strategy for enhancing photocatalytic ability. Specifically, reducing the bandgap of SnO_2_ will increase the photoresponse in the visible light region, making up 45% of the solar spectrum. Moreover, reducing the bandgap will also create many defect states that can decrease the recombination of photogenerated electron–hole pairs. There are many approaches to narrowing the bandgap of SnO_2_, such as modifying SnO_2_ by noble metal, graphene, or doping, including self-doping SnO_2_ (Sn^2+^-doped SnO_2_ or SnO_2−_*_x_*). In general, doping SnO_2_ will reduce the bandgap, which enhances the photoactivity in the visible light region for SnO_2_. The narrowing of the bandgap by introducing defects in metal oxide semiconductors opens up the possibility of their use in the visible spectrum [77]. Recently, Xie et al. reported using SnO_2_/graphene quantum dot (GQD) composites. They showed that the absorption edge of as-prepared SnO_2_ (Figure 13a black line) is around 340 nm, equaling to a bandgap of 3.64 eV. The PL peak of SnO_2_ was located in the range of 280–485 nm (Figure 13b). The combination of GQDs and SnO_2_ did not affect the shape of the PL peak. However, the corresponding PL intensity of the SnO_2_/GQDs sample was decreased because of the greatly reduced radiative charge recombination of SnO_2_. Moreover, enhanced visible light response and enhanced charge separation in the sample with GQDs have been observed (Figure 13c). The EIS measurements (Figure 13d) indicated that the diameter of the arc radius of SnO_2_/GQDs (1%) is much smaller than that of SnO_2_, confirming that the GQDs contributed to improving the charge separation, significantly reducing indoor NO under visible light irradiation. The optimized composite removed 57% of the initial NO while generating a negligible amount of NO_2_. In addition, this work found that the insertion of graphene quantum dots did not induce any noticeable impact on the structure of the SnO_2_ component. Still, its presence strongly enhanced energy harvesting and charge separation in the resulting composite [36].

**Figure 14:**
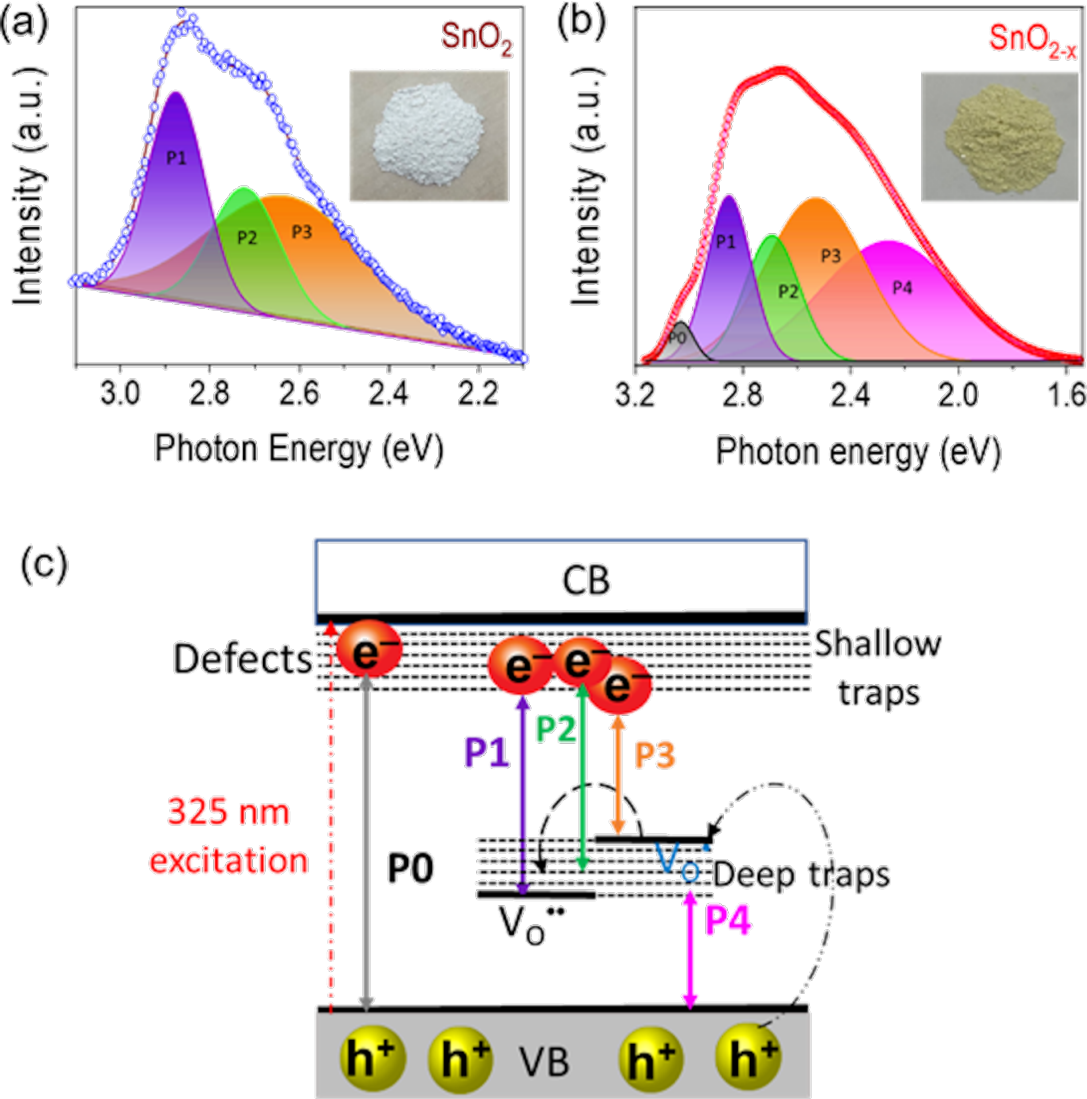
Gaussian fit of PL spectra with inserted images of sample color of SnO_2_ (a) and SnO_2−_*_x_* (b); and proposed schematic model for emissions from defects in SnO_2_ and SnO_2−_*_x_* (c). Figure 14 was reproduced from [73], © 2021 The Chinese Ceramic Society. Production and hosting by Elsevier B.V. This is an open access article under the CC BY-NC-ND license (http://creativecommons.org/licenses/by-nc-nd/4.0/). This content is not subject to CC BY 4.0.

Regarding the self-doping SnO_2_, Pham et al. reported on the fabrication of a SnO_2−_*_x_*/g-C_3_N_4_ heterojunction, inducing an S-scheme interface, showing impressive photocatalytic NO removal under visible light. In this work, Pham et al. indicated that deep trap centers of OV defects (Figure 14) formed with a very high concentration (36.69%), mainly from V_O•_ and V_O••_ centers. These OVs reduced the bandgaps of SnO_2_ (3.7 eV) and SnO_2−_*_x_* (3.17 eV), significantly impacting the reaction rate during the photocatalytic process, leading to enhanced NO removal under visible light. Also, the reported selectivity of the SnO_2−_*_x_*/g-C_3_N_4_ heterojunction is three times higher than that of the bare materials. The finding of this work further supports the importance of OVs in the design of photocatalytic materials [73].

**Figure 15:**
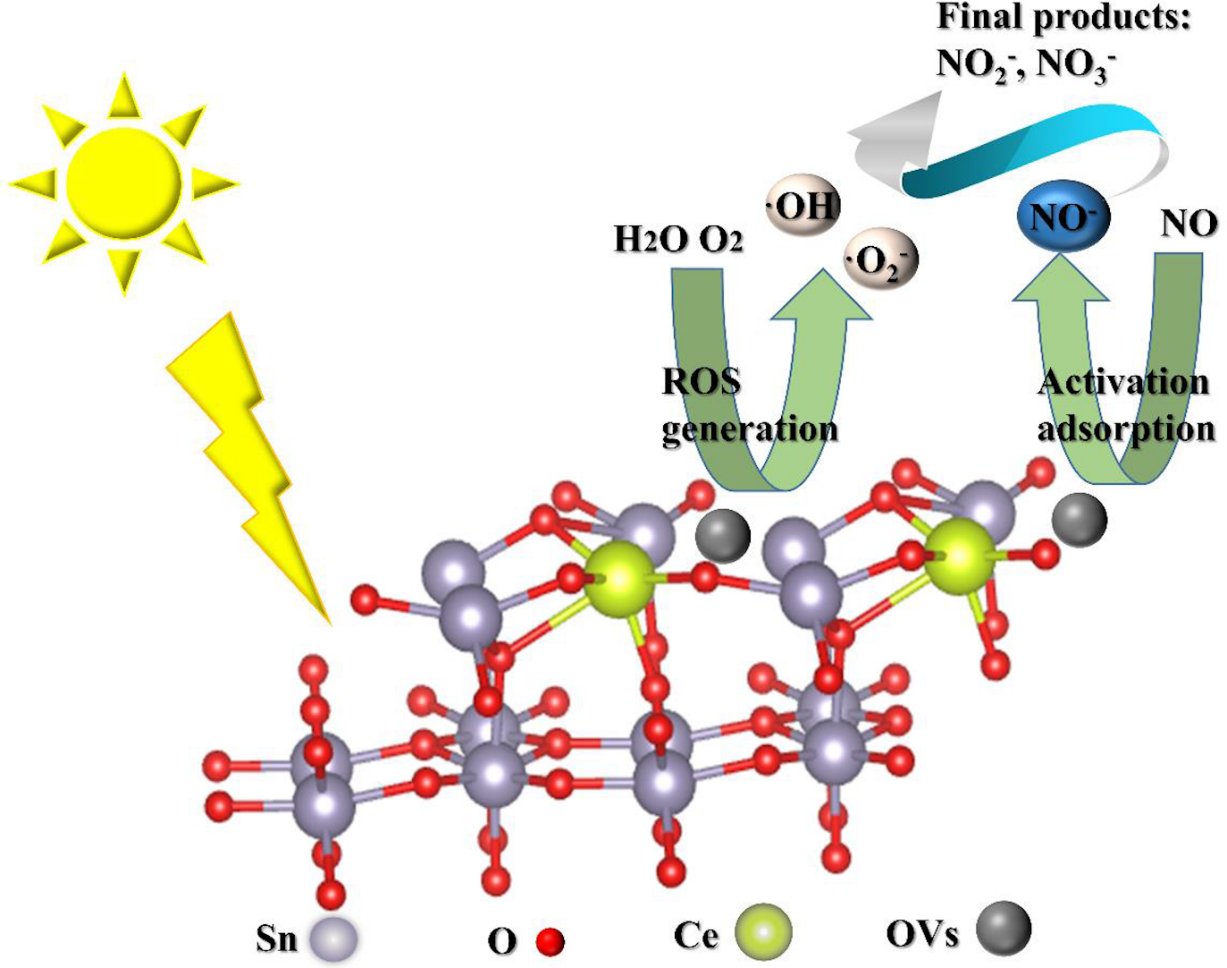
The proposed process of NO + O_2_ reaction over Ce–SnO_2_ under visible light irradiation. The ROS reacted with the activated NO^−^ intermediates to nitrates and nitrites. Figure 15 was reprinted from [39], Applied Catalysis B: Environmental, vol. 284, by Song, X.; Qin, G.; Cheng, G.; Jiang, W.; Chen, X.; Dai, W.; Fu, X. “Oxygen defect-induced NO− intermediates promoting NO deep oxidation over Ce doped SnO_2_ under visible light”, article no. 119761, Copyright (2020), with permission from Elsevier. This content is not subject to CC BY 4.0.

Song et al. synthesized Ce-doped SnO_2_ materials with a high number of OVs to improve NO oxidation removal efficacy (Figure 15). The results showed that the excellent NO oxidation activity of Ce–SnO_2_ materials was based on the OVs, which create a suitable site for the formation of NO^−^ intermediates to generate nitrite and nitrate products in the photocatalytic reaction processes. Moreover, additional OVs could be readily formed by thermal treatment under argon atmosphere. The work suggested an innovative approach for developing high-performance photocatalysts and a cost-effective, environmentally benign way through heat treatment in different atmospheres [39].

**Figure 16:**
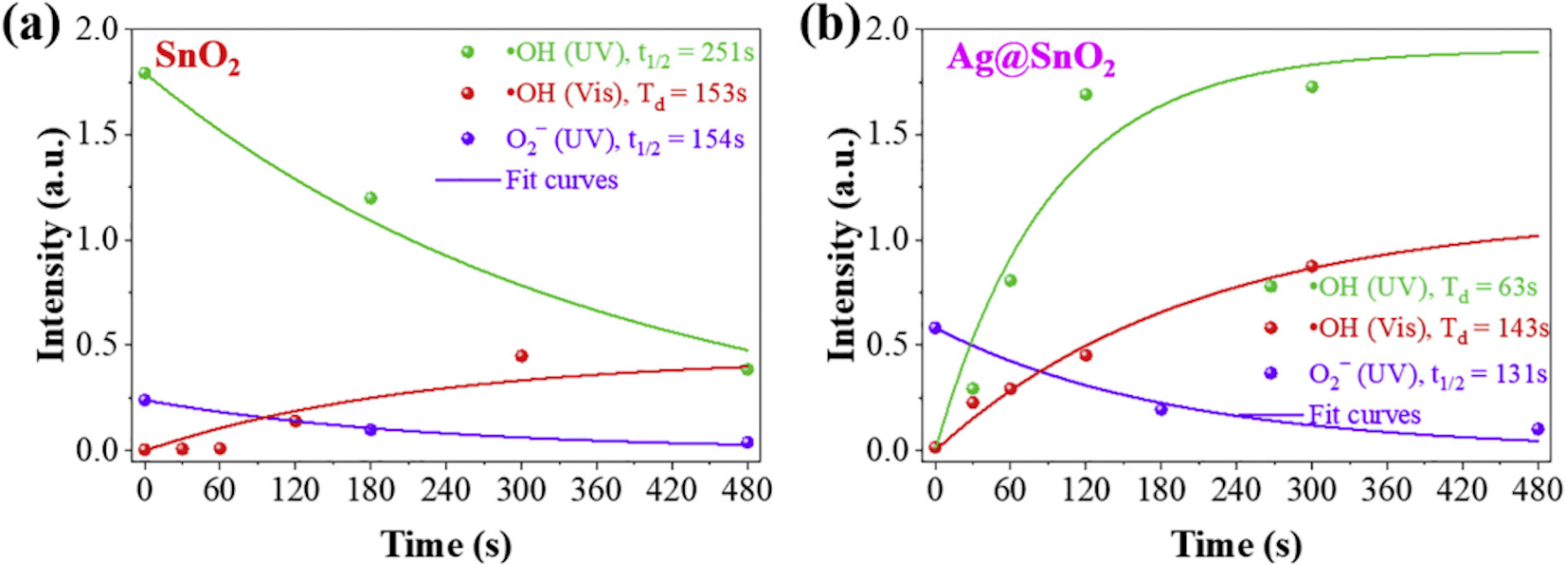
Decay and growth curves of primary ROS versus radiation time of SnO_2_ NPs (a) and Ag@SnO_2_ (b). Figure 16 was reprinted from [72], Catalysis Communications, vol. 136, by Bui, D. P.; Nguyen, M. T.; Tran, H. H.; You, S.-J.; Wang, Y.-F.; Van Viet, P. “Green synthesis of Ag@SnO_2_ nanocomposites for enhancing photocatalysis of nitrogen monoxide removal under solar light irradiation”, article no. 105902, Copyright (2019), with permission from Elsevier. This content is not subject to CC BY 4.0.

Combining noble metals with SnO_2_, such as in Au/SnO_2_ööö[78] or Pd/SnO_2_ööö[79], is an advanced approach yielding an effective performance for gas sensing. However, There is only one report by Bui et al. on using Ag@SnO_2_ NPs for removing NO, taking advantage of plasmonic-induced photocatalysis [72]. The Ag@SnO_2_ NPs were fabricated by a simple and green approach using hydrothermal growth and photoreduction deposition. The introduction of Ag induced a bending of the band structure of SnO_2_ NPs, leading to a change of the Fermi level. As a result, the Ag@SnO_2_ NPs showed an impressive photocatalytic NO removal of 70% while generating very little NO_2_ (4%) after 30 min. In addition, this work one to understand the underlying photocatalytic mechanism through the species lifespan obtained from trapping experiments and time-dependent ESR signals (Figure 16). Electrons and holes are equally important for photocatalysis [72].

## Conclusion

Regarding the improvement of the photocatalytic NO degradation over SnO_2_ nanomaterials there are many developments and approaches, such as BiOBr/SnO_2_, g-C_3_N_4_/SnO_2_, SnO_2_/NCDs/ZnSn(OH)_6_, Ce-doped SnO_2_, SnO_2_ self-doped with Sn^2+^, and Ag@SnO_2_. These systems yielded an enhanced photocatalytic NO*_x_* degradation either through increasing the charge transfer, through structural changes leading to bandgap reduction, or through the generation of favorable surface states for the NO*_x_* decomposition reaction. However, the performance in NO removal is still low (only nearly 60% under visible light and 75% under solar light). Also, the syntheses of the materials are difficult to upscale to an industrial scale. Moreover, the photocatalysts were prepared in powder form, which is not suitable for emerging applications. Based on this review, we suggest the following subjects for future research: (1) improving the NO photocatalytic degradation by combining other favorable bandgap semiconductors; (2) constructing a ternary heterostructure to create double Z-scheme/S-scheme materials, preferably using two redox sites; (3) synthesizing other morphologies of SnO_2_ such as nanorods, nanotubes, or 3D structures to increase the specific surface area of the catalyst; (4) upscaling the syntheses and using other synthesis approaches such as sol–gel or chemical vapor deposition to form thin film materials that can replace powder materials, (5) adhering the catalyst materials on commercial films such as polypropylene, polytetrafluorethylene, or PM2.5 films for real-life applications, such as air filters and NO*_x_* gas treatment membranes; and (6) applying the materials in biological media where the presence of NO/NO_2_ is predominant.
